# Diversity and Genome Analysis of Australian and Global Oilseed *Brassica napus* L. Germplasm Using Transcriptomics and Whole Genome Re-sequencing

**DOI:** 10.3389/fpls.2018.00508

**Published:** 2018-04-19

**Authors:** M. Michelle Malmberg, Fan Shi, German C. Spangenberg, Hans D. Daetwyler, Noel O. I. Cogan

**Affiliations:** ^1^AgriBio, Centre for AgriBioscience, Agriculture Victoria, Bundoora, VIC, Australia; ^2^School of Applied Systems Biology, La Trobe University, Bundoora, VIC, Australia

**Keywords:** nucleotide diversity, sequence-based genotyping, *Brassica napus*, RNA-Seq, genotyping-by-sequencing, variant annotation

## Abstract

Intensive breeding of *Brassica napus* has resulted in relatively low diversity, such that *B. napus* would benefit from germplasm improvement schemes that sustain diversity. As such, samples representative of global germplasm pools need to be assessed for existing population structure, diversity and linkage disequilibrium (LD). Complexity reduction genotyping-by-sequencing (GBS) methods, including GBS-transcriptomics (GBS-t), enable cost-effective screening of a large number of samples, while whole genome re-sequencing (WGR) delivers the ability to generate large numbers of unbiased genomic single nucleotide polymorphisms (SNPs), and identify structural variants (SVs). Furthermore, the development of genomic tools based on whole genomes representative of global oilseed diversity and orientated by the reference genome has substantial industry relevance and will be highly beneficial for canola breeding. As recent studies have focused on European and Chinese varieties, a global diversity panel as well as a substantial number of Australian spring types were included in this study. Focusing on industry relevance, 633 varieties were initially genotyped using GBS-t to examine population structure using 61,037 SNPs. Subsequently, 149 samples representative of global diversity were selected for WGR and both data sets used for a side-by-side evaluation of diversity and LD. The WGR data was further used to develop genomic resources consisting of a list of 4,029,750 high-confidence SNPs annotated using SnpEff, and SVs in the form of 10,976 deletions and 2,556 insertions. These resources form the basis of a reliable and repeatable system allowing greater integration between canola genomics studies, with a strong focus on breeding germplasm and industry applicability.

## Introduction

*Brassica napus* (2n = 4x = 38, AACC) is a recent allotetraploid originating from natural hybridization and genome duplication events between *Brassica rapa* and *Brassica oleracea*, sometime after *B. rapa* and *B. oleracea* diverged, between 12,500 and 7,500 years ago (Chalhoub et al., 2014) and appears to have arisen from multiple origins (Song and Osborn, 1992; Allender and King, 2010). Domestication of *B. napus* began relatively recently (400–500 years ago) and no truly wild populations have been recorded (Gómez-Campo and Prakash, 1999). Although swede and fodder varieties exist, it is primarily used as an oilseed crop with applications as a food source, lubricant, and biofuel. As breeding efforts within the last 60 years have specifically targeted erucic acid and seed glucosinolate content (Walker and Booth, 2001; Wu et al., 2008), and due to high oil and protein content, canola has become the world’s second most important oilseed crop after soy bean, especially in Canada, China, India, Europe, and Australia^1^.

Additional breeding efforts to adapt canola to local environments has further narrowed the gene pool and resulted in winter, spring, and semi-winter growth habits based on vernalization requirements, which is the primary factor affecting genetic differentiation and population structure in canola (Delourme et al., 2013; Li et al., 2014; Gazave et al., 2016). Within eco-geographic origins, cultivar relationships usually reflect breeding history, with some countries creating more isolation than others (Cowling, 2007; Wang et al., 2009; Qian et al., 2014).

Previous examinations of diversity between growth habits have found spring types to exhibit the highest level of nucleotide diversity, followed closely by winter types, with Chinese semi-winter varieties having the lowest (Bus et al., 2011; Delourme et al., 2013; Wang et al., 2014) and the least genetic differentiation between accessions (Wang et al., 2014), which is likely due to the isolation of Chinese varieties since the establishment of local breeding programs (Chen et al., 2008). Similarly, Gazave et al. (2016) found spring and winter types to harbor similar levels of diversity, and while they found Asian winter varieties to be the most diverse, in contrast to previous studies, the varieties used were largely composed of Japanese and South Korean varieties, with a small proportion of Chinese lines.

Although analyses of global *B. napus* germplasm showed spring types to be among the most genetically diverse, Australian spring germplasm is bottlenecked (Gyawali et al., 2013) due to intensive breeding efforts for increased blackleg disease resistance, reduced photoperiod requirements for flowering (Cowling, 2007) and dry climate tolerance (Walker and Booth, 2001). The majority of Australian cultivars released between 1995 and 2002 were derived from 11 ancestral varieties (five European or Canadian spring types, one *B. juncea*, and five Asian lines) and have remained isolated, with signs of loss of genetic diversity due to genetic drift as of the year 2000 (Cowling, 2007). Therefore, similar to Chinese germplasm, current Australian germplasm is likely to have low levels of diversity, with limited studies of Australian germplasm and would benefit from specific introgressions.

Although not as isolated, overall diversity in global oilseed germplasm is also narrow due to recent and intensive breeding, such that canola would benefit from genomics approaches to mitigate further erosion of diversity, and to exploit diversity in a targeted manner in breeding enhancement programs. While most commonly used to incorporate beneficial regions through selective breeding, genomic selection (GS) is able to use marker information to maintain diversity, and is a promising approach for the improvement of canola germplasm, while keeping genetic erosion in check (Meuwissen et al., 2001; Lin et al., 2017). As the method assumes causative mutations and sampled genetic markers are in linkage disequilibrium (LD), it is essential to saturate the genome with molecular markers, with optimal marker density dependent on a number of factors, such that a thorough understanding of diversity and LD within canola breeding populations is beneficial.

Just as estimates of diversity differ depending on sample composition, particularly in terms of eco-geographic origin, LD too has been found to vary substantially. While initial studies concluded that *B. napus* has low levels of overall LD, these same studies reported average LD to extend anywhere between 250 and 1000 kb (Ecke et al., 2010; Zou et al., 2010; Bus et al., 2011; Xiao et al., 2012; Delourme et al., 2013), a non-trivial degree of LD, as is expected in a species with low overall genetic diversity (Hasan et al., 2006). In contrast, for example, maize typically displays rapid decay of LD, reaching an *r*^2^ value of 0.2 within c. 1 kb (Romay et al., 2013). However, these *B. napus* studies were not optimal, using low marker density from mostly PCR-based markers or small sample size.

A few studies using a higher density of single nucleotide polymorphism (SNP) markers (c. 10–25K) also report varying levels of LD, with considerable variation between individual chromosomes, and on average less rapid LD decay in the C genome (Qian et al., 2014; Wang et al., 2014; Wu et al., 2016). Notably, examining LD in diverse canola varieties versus predominantly sampling a single eco-geographic group significantly affected estimated LD, dropping by 60% from a whole genome estimate of 1,214 kb (*r*^2^ = 0.26) in Chinese varieties (Wu et al., 2016) to 490 kb (*r*^2^ = 0.2) across a diverse sample set (Wang et al., 2014). This has also been observed in maize, with tropical germplasm exhibiting typical LD of c. 5–10 kb, while for temperate germplasm this extends to c. 10–100 kb when *r*^2^ = 0.1 (Lu et al., 2011). As such, population structure can have a significant effect, and comprehensive understanding of LD within breeding programs is necessary for the effective application of GS using marker dense genotyping.

Reduced representation genotyping methods have been valuable in cheaply generating SNP markers, allowing large sample sets to be genotyped, and enabling the application of GS and other crop improvement schemes. However, these systems are not without issues and can be sub-optimal, including the commonly used genotyping-by-sequencing (GBS) through restriction-site associated DNA (GBS-RAD) method (Elshire et al., 2011) and variations thereof, such as double digestion RAD (ddRAD; Peterson et al., 2012; Poland et al., 2012), which has issues with high missing data and dominant markers. The other commonly used genotyping method in canola is the *Brassica* 60K SNP array, which does not allow for novel SNP discovery and any genetic diversity inferences made based on predefined SNPs must be aware of the bias caused by the initial SNP discovery method. The 60K *Brassica* SNP array also requires the removal of a significant number of markers due to poor alignment, by excluding SNPs whose flanking sequences map to multiple sites in the reference genome (Mason et al., 2017). One study using the *Brassica* 60K SNP array found chromosome C07 to display almost no LD, which is likely a result of mapping error and low SNP number due to discovery bias (Wang et al., 2014).

Furthermore, many of these systems fail to provide a common standard and platform to integrate studies for maximal benefit, particularly in the case of GBS-RAD and older systems such as AFLPs and SSRs. With the release of a reference genome for canola, the establishment of genomic resources which can be reliably integrated and reproduced between studies and datasets has become possible, and should be pursued. Whole genome re-sequencing (WGR) allows for a greater level of genome interrogation than complexity reduction methods and results in a significant increase in SNP markers. A well-curated list of SNPs with predicted effects, based on the reference genome positions will assist in driving breeding forward through evaluation of germplasm. To date, significant work in this area has been done by Huang et al. (2013) who found 892,803 SNPs by sequencing the genomes of 10 canola varieties, representing a small portion of total global diversity and Schmutzer et al. (2015), who found 4.3 million SNPs from 52 varieties that covered the diversity of *B. napus* as well as re-synthesized lines. However, only 25 of these varieties were oilseed types, with the rest comprising re-synthesized lines, vegetable, swede, and fodder varieties. The oilseed varieties used by Schmutzer et al. (2015) are mostly European winter varieties, one European spring type, and two Asian varieties. As of yet, no study has used a substantial representation of global *B. napus* oilseed breeding germplasm to develop a foundation of high-quality SNPs to base genome studies from, which is also of high industry relevance.

WGR affords the opportunity to interrogate polymorphisms other than SNPs, including structural variants (SVs) (>50 bp), which may be the cause of functional genetic differentiation and potential sources of heterosis. SVs are particularly relevant as all plant species have undergone whole genome duplication events followed by diploidization (Wendel, 2000), and there has occurred a *Brassiceae*-specific whole genome triplication (Lysak et al., 2005), resulting in gene loss and neo-/sub-functionalization such that a *B. napus* gene is represented an average of c. 4.4 times in the whole genome (Parkin et al., 2010), ranging from 1 to 12 (Schiessl et al., 2014).

This study aimed to generate the most comprehensive genome analysis of canola global germplasm to date that is lacking from the literature. As recent studies have focused on European and Chinese varieties, this study selected a significant number of Australian spring types as well as diverse global germplasm, which was initially interrogated for population structure using GBS-transcriptomics (GBS-t), which provides reduced representation of the genome for sequencing by extracting and converting mRNA to RNA-Seq libraries, allowing for cost effective genotyping of a large number of samples (Malmberg et al., 2017). As a result of this assessment, a core collection of samples representative of global diversity was used for WGR, where DNA is extracted and the whole genome is re-sequenced, resulting in a higher cost per sample but providing more genomic information. Both the GBS-t and WGR data sets were used for a side-by-side comparison of population structure, diversity, and LD, and to establish the validity of a reduced representation method in canola. The whole genomes were further used to develop genomic resources for the improvement of canola germplasm, including a list of high-confidence SNPs with annotations and effects predicted, and a set of deletions and insertions as an initial exploration of SVs in canola. This data will provide a strong basis for future canola genomics work, with a focus on canola breeding industry relevance.

## Materials and Methods

### Plant Material

Seed was sourced from the Australian Grains Genebank and Denise Barbulescu, National *Brassica* Germplasm Improvement Program, Grains Innovation Park, Agriculture Victoria Research, Victoria, Australia.

#### GBS-Transcriptomics

GBS-t raw sequencing data of 540 samples from Malmberg et al. (2017) were re-analyzed with the addition of 93 new samples which were sequenced following the same pipeline as previously described, resulting in a total of 633 samples, representing 627 canola varieties from 27 countries. These include 258 Australian samples, 271 European samples, 69 Asian samples, 8 North American samples, 2 New Zealand samples, 1 African sample, and 24 samples of unknown origin (full details provided in **Supplementary Table S1**).

#### Whole Genome Re-sequencing

Of the 633 samples genotyped using the GBS-t method, 149 samples were processed for WGR, including one biological replicate (**Supplementary Table S1** and **Supplementary Figure S1**). This included 10 samples comprising both Australian spring types and diverse winter types that were used in experimental development of WGR library preparation, 6 commonly studied European winter types, 87 significant Australian spring types, and 46 samples forming a global diversity panel chosen using a custom pipeline, selecting a representative of each branch clade from a neighbor-joining (NJ) tree, to capture overall diversity (Shi et al., 2017).

### Library Preparation and Bioinformatic Data Analysis

#### GBS-Transcriptomics

Library preparation of the 93 additional samples was performed according to the methods described in Malmberg et al. (2017). Briefly, mRNA was extracted using a Dynabead method (Life Technologies, Carlsbad, CA, United States) and RNA-Seq libraries generated using the SureSelect stranded RNA library preparation kit (Agilent Technologies, Santa Clara, CA, United States). Around three million sequencing reads were generated per sample and after read quality trimming and alignment to the Darmor CDS reference genome (Chalhoub et al., 2014), SNP genotyping of all 633 samples was performed according to the methods described in Malmberg et al. (2017), using the 226,855 high quality SNP loci previously generated by Malmberg et al. (2017), as well as filtering for a minimum mapping quality of 30.

#### Whole Genome Re-sequencing

Genomic DNA was extracted from young leaf tissue using the DNeasy 96 Plant Kit (QIAGEN, Hilden, Germany), according to the manufacturer’s instructions. Two methods of whole genome library preparation were evaluated in 10 samples for sequence coverage and ease of scaling: the Illumina TruSeq PCR-free kit (San Diego, CA, United States) using physical shearing using the S2 focused-ultrasonicator system (Covaris, MA, United States) and an enzymatic MspJI (NEB, MA, United States) shearing method after the introduction of 5-methyl-dCTP (NEB) described in Shinozuka et al. (2015). Methylated C was incorporated into the genome using the REPLI-g mini kit (QIAGEN), following the manufacturer’s instructions, with the addition of 600 μM 5-methyl-dCTP. The amplified DNA was digested with MspJI following manufacturer’s instructions. The digested product underwent an end-filling and dA-tailing reaction using Klenow Fragment (NEB), followed by adapter ligation using in-house adapters for the preparation of Illumina sequencing libraries. The adapter ligated libraries were purified using Agencourt AMPure XP beads (Beckman Coulter, Pasadena, CA, United States) at equal volumes. Final libraries were produced by performing a PCR with in-house barcoded primers and Phusion Hot Start DNA polymerase (Thermo Fisher Scientific, Waltham, MA, United States). Final libraries were cleaned with AMPure XP beads at a ratio of 1:0.8 volumes of sample to beads.

Quantification of libraries for the purpose of pooling was performed using an Illumina MiSeq Nano 300 cycle run based on the method described in Katsuoka et al. (2014). Samples were pooled based on percentage reads as a relative unit and subsequently sequenced on an Illumina HiSeq3000 aiming for 10× read depth coverage of each sample. The full sequence data for all 149 samples have been deposited with links to BioProject accession number PRJNA435647 in the NCBI BioProject database^2^.

Read mapping and SNP discovery was based on the Malmberg et al. (2017) method described for canola, with the exception of aligning reads to the whole genome reference (Chalhoub et al., 2014) and filtering for a minimum mapping quality of 30. Briefly, samples were grouped into Australian spring types and the global diversity panel before undergoing SNP discovery using SAMtools mpileup (Li et al., 2009). The resulting SNPs from each group were separately filtered for a minimum read depth of 5, maximum missing data of 50%, minimum minor allele frequency (MAF) of 5% and maximum heterozygosity of 40%. The resulting SNPs were consolidated to produce a preliminary list of unique SNPs which was used as a SNP list to re-process all BAM files with SAMtools mpileup, creating a complete SNP profile for all samples and SNP loci.

#### SNP Filtering of Pre-discovered Variant Sites

For both the GBS-t and WGR data, the VCF file resulting from initial processing through SAMtools mpileup with the appropriate SNP list was further filtered in R (R Development Core Team, 2012) for a minimum read depth of 5, maximum missing data of 50%, minimum MAF of 5% calculated separately for Australian spring types and the global diversity panel, and maximum heterozygosity of 10% (**Supplementary Methods S1** and **Supplementary Figure S2**).

#### Nei’s d Neighbor-Joining Tree and AMOVA

For each of the high-confidence SNP sets resulting from the GBS-t and WGR data, Nei’s pair-wise genetic distance was calculated for all samples and subsequently, genetic diversity within canola was calculated using StAMPP (Pembleton et al., 2013). NJ trees were generated based on the method proposed by Saitou and Nei (1987) and displayed in DARwin v6.0.5 (Perrier and Jacquemoud-Collet, 2006).

To determine the percentage of variation caused by between- and within-population differences, StAMPP was used to perform an analysis of molecular variance (AMOVA; Excoffier et al., 1992). Due to greater sample size, the GBS-t data was used for this analysis. Samples were grouped based on geographic origin to form three populations encompassing Australian, European, and Asian varieties, respectively. Samples from other geographic regions, including New Zealand, North America, and Africa, and those of unknown origin were excluded from this analysis due to insufficient sample size.

#### Linkage Disequilibrium

Additional filtering was performed in order to evaluate LD. The GBS-t samples were again grouped into three populations representing Australian, European, and Asian samples, with the exclusion of other regions due to small sample size. The c. 226K list-based SNP profiles of each population were filtered for read depth of 5, maximum missing data of 40%, minimum MAF of 0.1 across all samples and maximum heterozygosity of 10%, as well as removing any SNPs without a fixed position in the reference genome. LD was evaluated for each population using *r*^2^ which was derived from pair-wise comparisons using PLINK (Purcell et al., 2007). The same SNP filtering parameters were applied to the WGR samples as a single population and each chromosome processed individually in PLINK up to 5 Mb. Pair-wise *r*^2^ values were binned according to an expanding graduated distance scale, averaged in each bin and plotted in R.

#### SNP Annotation

The resulting high-confidence SNP set from the WGR data was annotated using SnpEff (Cingolani et al., 2012). The SnpEff binary database file (.bin) was generated using the *B. napus* Darmor-*bzh* genome annotation file v5 (gff3) and the whole genome reference sequence (Chalhoub et al., 2014).

#### Structural Variant Discovery

After initial quality control by Breakdancer (Chen et al., 2009), 15 samples were excluded from further analysis as the coefficient of variation of the insert size exceeded the cutoff value of 1. As such, 134 samples had sufficiently high quality coverage for SV discovery and were processed with Breakdancer and Pindel (Ye et al., 2009), filtering for a minimum read depth of 5. Due to the highly duplicated allotetraploid nature of the *B. napus* genome, reliable detection of duplications and inter-chromosomal translocations, as opposed to homoeologous regions, is difficult with short-read sequencing technology and necessitates long-read sequencing. In order to confidently detect SVs in the current data set, only deletions and insertions were examined. For deletions, only SVs that were identified by both programs were kept. Insertions were only identified by Pindel. For both SV classes, individual data was combined using BEDTools multiinter (Quinlan and Hall, 2010), indicating regions of structural variation in the sample set, and further filtered in R for a minimum length of 50 bp and minimum MAF of 0.05 across all samples. SVs were annotated using the *B. napus* Darmor-*bzh* genome annotation file v5 (gff3; Chalhoub et al., 2014).

## Results

### Evaluation of Population Structure Using GBS-Transcriptomics

**FIGURE 1:**
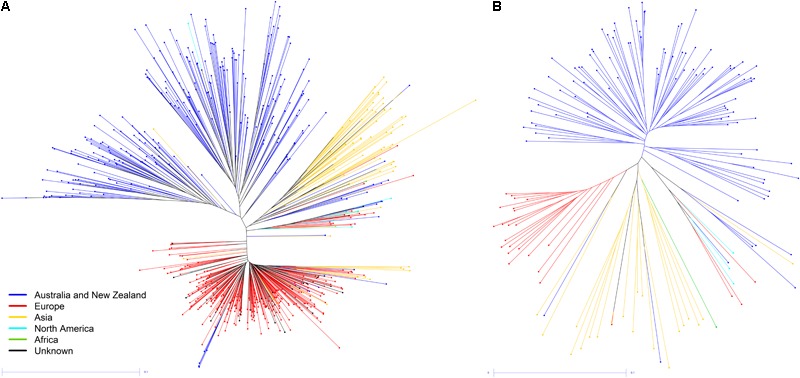
NJ tree calculated using Nei’s pairwise genetic distance for **(A)** GBS-t data set of 633 samples and **(B)** WGR data set of 149 samples.

The average coverage of the CDS reference genome, excluding two samples with extremely high coverage (>100×), was 8.3× per sample. After genotyping all 633 GBS-t samples using the 226,855 SNP list, these sites were additionally filtered in order to only retain SNPs which were polymorphic and passed quality filtering in this sample set, resulting in 61,037 informative SNPs. To assess population structure, Nei’s genetic distance was calculated and an NJ tree generated (**Figure 1A**). Clustering revealed population structure broadly subdivided based on growth habit with spring, winter, and semi-winter groups, and was confirmed by analysis with STRUCTURE (Pritchard et al., 2000: **Supplementary Methods S1** and **Supplementary Figure S3**). Clustering is also largely consistent with geographic origin as the majority of Australian varieties in the data sets are spring types, European varieties are winter types and Asian lines have a large proportion of Chinese semi-winter types as well as other Asian winter types. AMOVA revealed that 37.1% (*P* = 0.000) of total variation could be attributed to differences between populations and 62.9% is due to differences between individuals. From the NJ tree, a representation of global *B. napus* germplasm diversity was selected for WGR, as well as a significant proportion of Australian spring type lines (**Figure 1B** and **Supplementary Figure S1**).

### Whole Genome Re-sequencing Library Preparation and SNP Discovery

Initial analysis of 10 samples processed using the Illumina TruSeq PCR-free kit using covaris shearing and the MspJI based enzymatic shearing method resulted in similar coverage of the genome (**Supplementary Figure S4**), and due to the ease of scaling for high-throughput library preparation, the enzymatic method was used to process all remaining samples.

**FIGURE 2:**
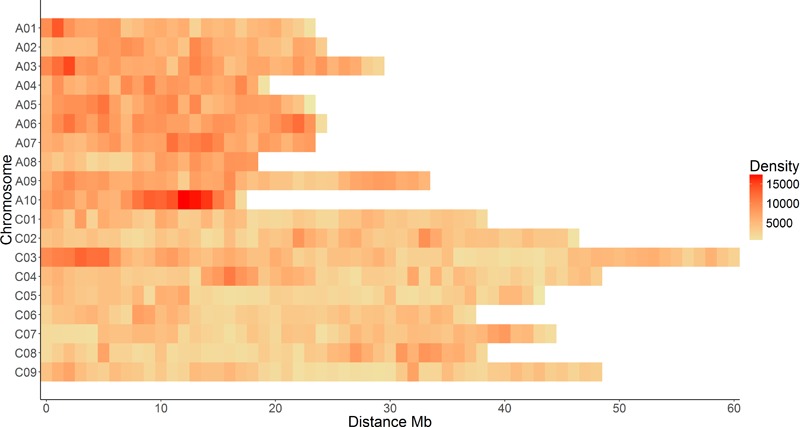
Distribution of c. 4 million WGR SNPs in 1 Mb bins across individual chromosomes, excluding unanchored sequences represented in “random” scaffolds of the reference genome.

The shotgun sequencing of all 149 samples generated c. 8 billion reads with an average read length of 147 bp, resulting in an average genome coverage of 9.27× per sample. To reduce the computational burden, *de novo* SNP discovery was performed separately in Australian spring types and the global diversity panel. Initial filtering resulted in 6,163,261 and 7,562,468 SNPs respectively. Of these, 9,494,358 were unique and biallelic across all samples and were subsequently used as a lenient SNP list, as these have only been filtered on maximum heterozygosity of 40%. Further stringent filtering across all samples while still calculating MAF separately, resulted in 4,029,750 high-confidence SNPs (**Table 1, Figure 2** and **Supplementary Table S2**). SNP density differed significantly between sub-genomes, with a base change occurring, on average, every 141 bp and 264 bp on the A and C genomes respectively (**Table 1**).

**Table 1:** Distribution of high-confidence SNPs from 149 WGR samples, excluding chromosome sequences with a “random” designation in the Darmor-*bzh* whole genome reference.

Chromosome	SNPs	Chromosome size (bp)	Mean distance between SNPs
A01	139,635	23,267,856	167
A02	148,998	24,793,737	166
A03	214,265	29,767,490	139
A04	141,057	19,151,660	136
A05	172,456	23,067,598	134
A06	201,894	24,396,386	121
A07	181,320	24,006,521	132
A08	104,516	18,961,941	181
A09	209,826	33,865,340	161
A10	181,275	17,398,227	96
A genome mean	169,524	23,867,676	141
C01	148,571	38,829,317	261
C02	181,409	46,221,804	255
C03	292,068	60,573,394	207
C04	192,990	48,930,237	254
C05	129,711	43,185,227	333
C06	142,533	37,225,952	261
C07	159,915	44,770,477	280
C08	142,288	38,477,087	270
C09	152,244	48,508,220	319
C genome mean	171,303	45,191,302	264
Whole genome mean	170,367	33,968,341	199

The whole genomes were used comparatively with the GBS-t global data set to confirm population structure and to evaluate diversity and LD. The WGR data was further used for annotating SNP effects and identifying SVs.

### Parallel Evaluation of Global Canola Germplasm Using GBS-t and WGR

#### Population Structure of WGR Lines in Comparison to GBS-t

The WGR NJ tree (**Figure 1B**) illustrated good consistency and reproducibility with the GBS-t data (**Figure 1A**). In both NJ trees, there is clear differentiation based on growth habit and also geographic origin. Breeding history and geographic isolation has impacted population structure, with Australian spring samples forming a cluster separate from several Canadian and European samples, initially misclassified as winter types but which were subsequently found to be spring types. Similarly, the majority of Chinese varieties group in the same clade, distinct from other Asian varieties.

#### Population Exclusive SNPs

Of the 61,037 high-confidence SNPs from the GBS-t data, 3,657 were only found in the global diversity panel and 519 were exclusive to Australian spring types (**Table 2**). For the c. 4 million WGR high-confidence SNPs, these figures were 593,742 and 203,752, respectively. In both instances, the majority of global diversity panel exclusive SNPs were located on the A genome while the majority of Australian spring exclusive SNPs were on the C genome (**Table 2**). Due to limited sampling it is not possible to preclude the presence of these exclusive SNPs in other varieties across sub-populations, but are nonetheless likely indicative of regions associated with selection or drift within sub-populations.

**Table 2:** Distribution of SNPs exclusive to Australian spring types or to the global diversity panel.

Sub-genome	GBS-t		WGR	
	Australian spring types	Global diversity panel	Australian spring types	Global diversity panel
A	160	**2,348**	94,175	**332,588**
C	**359**	1,309	**107,885**	257,534
U	0	0	1,692	3,620
Total	519	3,657	203,752	593,742

Total SNPs	61,037		4,029,750	

*The numbers highlighted in bold indicate the sub-genome bias within each population.*

#### Linkage Disequilibrium

**FIGURE 3:**
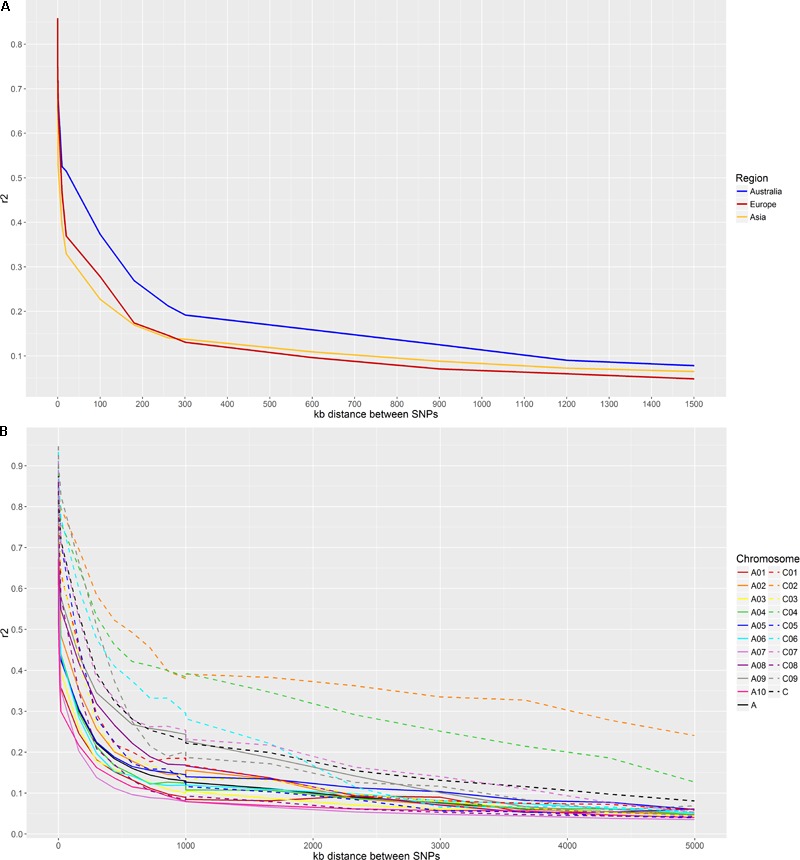
**(A)** LD decay based on GBS-t data. LD is examined in three major growing regions: Australia, Europe, and Asia. **(B)** LD decay based on WGR data for individual chromosomes. The A genome chromosomes are represented by solid lines and the C genome chromosomes are represented by dashed lines. The discontinuity at 1000 kb is an artifact of the binning process.

Due to the larger sample size, the GBS-t data was used to evaluate LD between geographic populations. From the c. 226K SNP list, informative SNPs which are variant within each sub-population were retained for LD analysis with 12,394 SNPs segregating in Australian varieties, 13,422 in Asian varieties and 8,080 in European varieties. LD decays the least rapidly in Australian varieties, reaching c. 280 kb when *r*^2^ = 0.2 and c. 1,100 kb when *r*^2^ = 0.1 (**Figure 3A**). In the short range, LD decays more rapidly in Asian varieties (c. 130 kb, *r*^2^ = 0.2) compared to European varieties (c. 160 kb, *r*^2^ = 0.2), but long range LD remains higher in Asian than European varieties (c. 700 kb and c. 550 kb, respectively, *r*^2^ = 0.1).

The higher SNP density provided by the WGR data was used to examine LD on a chromosome-by-chromosome basis, using 1,256,708 SNPs across the whole genome. LD varied extensively between individual chromosomes, with lowest LD decay observed for chromosome C02 and the fastest decay for A07 (**Figure 3B**). LD extended to c. 700 kb across the whole genome, c. 380 kb in the A genome and c. 1,600 kb in the C genome when *r*^2^ = 0.2.

### Development of Genomic Resources Using WGR

#### SNP Annotation

**FIGURE 4:**
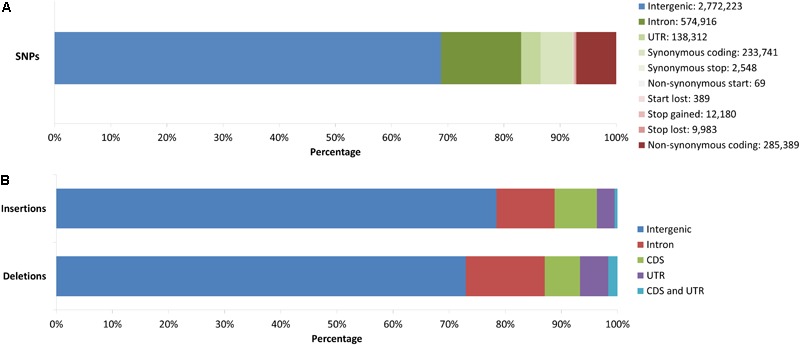
**(A)** Annotation of c. 4 million WGR discovered high-confidence SNPs using SnpEff. Green regions represent non-coding genic regions and synonymous substitutions, while red regions represent non-synonymous substitutions and the loss or gain of stop/start codons. **(B)** Annotation of structural variants in 134 samples using the Darmor-*bzh* gff3 file. Deletions and insertions were annotated based on overlap with intergenic regions or genic regions, sub-divided into intron, CDS, and UTR, and overlapping both CDS and UTR.

In order to assess the potential effect of SNPs, the c. 4 million high-confidence SNPs from the whole genomes were annotated using SnpEff (**Supplementary Table S2**). The majority of SNPs (68.8%) were found to be intergenic (**Figure 4A**). Of the 1,257,527 genic SNPs, 45.7% were intronic, with only a small percentage (0.3%) of these SNPs causing a splice site acceptor or donor region to occur, 11% were located in untranslated regions (UTRs), 18.8% were synonymous, 22.7% non-synonymous, and 1.8% of genic SNPs caused changes to stop codons or lost start codons (**Supplementary Table S2**). Transitions were more common than transversions with 57% and 43% respectively. Of the 101,040 genes described in the Darmor-*bzh* reference, 76,419 contained at least one SNP.

#### Structural Variants

The average number of SVs identified by Breakdancer and Pindel, as well as the number of unique SVs remaining after each filtering step is provided in **Supplementary Table S3**. Analysis of SVs in canola revealed that deletions were more common than insertions when compared to the reference, with 10,976 deletions affecting 2,583 genes and 2,556 insertions affecting 528 genes, of which 73% and 78.4% were intergenic, respectively (**Figure 4B**). Of the genic deletions, 52.1% were intronic, 23.2% were in CDS regions, 18.6% in UTRs and 6% spanned both CDS regions and UTR. For insertions, the same values were 48.1%, 34.8%, 14.7%, and 2.4%, respectively.

Deletions and insertions were evenly spread throughout the genome and there was not a higher prevalence in either sub-genome, although broadly within sub-genome, the number of SVs correlated with chromosome length. The smallest chromosome, A10, had the least number of deletions and insertions while A03 had the most deletions and C03, the largest chromosome, had the most insertions (**Table 3**). The deletions ranged in size from 50 bp to 15,420 bp with a median of 88 bp, and insertions were smaller, ranging from 50 bp to 132 bp with a median of 78 bp (**Supplementary Table S4**).

**Table 3:** Distribution of deletions and insertions >50 bp, identified across 134 WGR samples compared to the Darmor-*bzh* whole genome reference.

Chromosome	Deletions	Insertions
A01	344	81
A02	682	96
A03	1,059	146
A04	271	84
A05	233	79
A06	331	66
A07	553	90
A08	217	77
A09	696	108
A10	152	50
C01	450	113
C02	466	80
C03	787	164
C04	433	117
C05	193	72
C06	382	103
C07	352	93
C08	274	96
C09	284	111
Random scaffolds	2,817	730
Total	10,976	2,556

A small number of SVs were only found in one sub-population, with 139 Australian spring exclusive and 212 global diversity panel exclusive deletions, while for insertions, 14 were Australian spring exclusive and 91 were exclusive to the global diversity panel. Both deletions and insertions are generally more common in the global diversity panel, with an average of 11% of Australian spring samples and 13% of global diverse samples likely to harbor any given deletion, and for insertions the same figures were 12% and 19%, respectively.

## Discussion

Evaluation of the NJ trees revealed that a phylogeny based method is suitable for the classification of population structure, as was confirmed by the results of STRUCTURE (**Supplementary Methods S1** and **Supplementary Figure S3**), and provisional correction of growth habit. Phenotypic evaluation is necessary for conclusive re-classification; however, a phylogenetic approach is sufficient for growth habit grouping. As gene banks must rely on the information provided upon deposit, issues with misclassification of accessions have been observed (Hasan et al., 2006), and an initial examination of our data suggested this was the case for some samples. For instance, a small cluster in the GBS-t NJ tree, between European winters and Asian semi-winters contained, among others, AGG90078 which is the Canadian summer rape cultivar, Tribute (Rakow and Downey, 1993), and AGG95451 which is the Canadian cultivar Oro, the first low erucic acid summer rape (Fu and Gugel, 2010), but which were recorded as winter types in gene bank records. This led to a re-classification of growth habit in some of the varieties used in this study, based on the results of the NJ trees (**Supplementary Table S1**). The re-classified samples consistently cluster as expected based on breeding history and growth habit information found in the canola literature. As such, it is unlikely that the misclassifications found in this study are due to incorrect accession labeling of seed packets or the presence of an erroneous seed in the packet. Subjective attribution of growth habit may have affected the validity of passport data for some accessions.

Furthermore, a phylogeny based classification of population structure accounts for the combined effects of growth habit and recent breeding history. Despite being one of the original accessions used to establish Australian germplasm (Cowling, 2007), AGG95451 (Oro) shows clear differentiation from the majority of Australian spring varieties, instead clustering with other Canadian and European spring varieties, which were initially misclassified as winter types in gene bank records, forming a cluster distinct from Australian spring types. Conversely, while the majority of Chinese semi-winter samples group together, the Chinese winter type AGG96011 (Shen-Li Jutsaj; Wang et al., 2011), clusters with European winters, suggesting a winter type genetic background, with a greater impact on differentiation than eco-geographic origin, potentially due to a large proportion of European winter type genetic material in this variety. Overall, population structure was consistent with the findings of previous studies (Hasan et al., 2006; Bus et al., 2011; Delourme et al., 2013; Li et al., 2014; Gazave et al., 2016).

Recognition of population structure is vital for genomics based research as Fikere et al. (unpublished) showed improved prediction accuracy for traits relating to blackleg disease resistance, and Jan et al. (2016) saw improvement in the prediction of testcross performance in canola, when population structure was accounted for. Population structure information further assists with the utilization of diversity in breeding schemes, as it has been suggested that crossing plants of the same growth habit but displaying significant diversity is more valuable for the exploitation of heterosis than using varieties from a different growth habit due to the poor performance of canola between environments (Shi et al., 2011), and has the additional benefit of avoiding the need to perform backcrosses to restore the desired growth habit. The role of geographic origin in differentiation was confirmed by AMOVA, with a significant proportion of molecular variation attributable to between-population variation (37.1%). Nonetheless, the majority of variation is due to within-population differences (62.9%), highlighting the presence of significant variation between cultivars from the same region.

As anticipated, the global diversity panel had more SNPs found during initial SNP discovery based on WGR, more exclusive high-confidence SNPs, deletions and insertions, and greater likelihood of harboring any given SV. While the higher diversity observed in the global diversity panel compared to Australian spring types was expected due to the difference in sample composition, the moderate degree of diversity still present within Australian spring types was surprising. Despite previous findings of low overall diversity due to an isolated breeding history (Cowling, 2007; Chen et al., 2008), initial SNP discovery of the WGR data yielded 6,163,261 polymorphic Australian spring SNPs after the removal of monomorphic SNPs using minimum MAF filtering (0.05), compared to 7,562,468 SNPs in the global diversity panel. However, a relatively high number of polymorphic loci is expected as there were more Australian spring samples than in the global diversity panel (*n* = 94 and *n* = 55, respectively) and the reference genome is a winter type, such that diversity within Australian springs can still be considered low overall compared to global germplasm.

The other important finding from this evaluation was the degree of diversity present in the Asian population in this study, which may account for a large proportion of diversity observed in the global diversity panel. This analysis was based on the GBS-t data due to a larger sample size, allowing for evaluation of three major growing regions: Australia, Europe, and Asia. Despite the smallest population size, the Asian population retained the most polymorphic SNPs for LD analysis, suggesting a relatively high degree of diversity, and is likely due to the inclusion of non-Chinese Asian varieties. Although Chinese varieties have low levels of diversity due to an isolated breeding history (Chen et al., 2008; Bus et al., 2011; Wang et al., 2014), other geographic regions such as Japan, South Korea, India, and Pakistan have been found to harbor higher levels of diversity (Chen et al., 2008; Gyawali et al., 2013). Our findings support that of Gazave et al. (2016) who also genotyped diverse Asian varieties, primarily from South Korea and Japan, suggesting other Asian varieties may be a valuable source of diversity for European, Australian, and Chinese breeders.

The effect of population structure on LD has also been observed, with LD signatures between sub-populations of wheat and maize having been found to vary significantly (Lu et al., 2011; Voss-Fels et al., 2015), as was also clear in this study. Australian varieties exhibit the least LD decay of the three regions examined, consistent with low diversity and an isolated breeding history, while the Asian and European sub-populations displayed more rapid decay of LD due to higher diversity as well as low average MAF in the European varieties. While several studies have previously characterized LD in canola, recent studies have been largely, if not exclusively, composed of Chinese varieties (Qian et al., 2014; Wang et al., 2014; Wu et al., 2016), which are expected to display relatively low LD decay, and none have made comparisons between major germplasm pools. The significant variation between sub-populations highlights the value of examining LD within sub-populations to ensure appropriate marker density for association studies. As such, a highly diverse population will likely display more rapid decay of LD than estimated in this study, and consequently greater marker density will be required.

The evaluation of LD in genomes saturated with markers is particularly valuable for the evaluation of long-range LD, as low SNP density can cause artificial reduction of LD decay, which does not accurately reflect the true extent of LD. For instance, Wang et al. (2014) found almost no LD decay on chromosome C07 and attributed this to inaccurate SNP mapping. In this study, over 1 million SNPs across the genome were used to assess LD in individual chromosomes. There does not appear to be any clear pattern between chromosomes, though LD generally extends further in the C genome, as has been found in some studies (Qian et al., 2014; Wang et al., 2014; Wu et al., 2016). Sub-genomic LD in this study (380 kb on the A genome, 1,600 kb on the C genome, *r*^2^ = 0.2) falls between that found by Wang et al. (2014: 210 kb on the A genome, 810 kb on the C genome, *r*^2^ = 0.2) and Wu et al. (2016: 405 kb on the A genome, 2,111 kb on the C genome, *r*^2^ = 0.26), and is consistent with the diversity of the populations used in each study. Due to the strong influence of population structure, diversity, and other factors such as MAF on estimates of LD decay, a conservative interpretation of LD estimates is recommended and needs to be considered within the appropriate context.

Due to the highly duplicated nature of the canola genome, quality filtering to remove false positive SNPs is vital and while the most commonly used method involves using a BLAST against the reference genome to remove SNPs whose flanking sequences map to multiple locations, as is common practice when using the *Brassica* 60K SNP array (Li et al., 2014; Qian et al., 2014; Hatzig et al., 2015; Jan et al., 2016; Mason et al., 2017), this method is not optimal in a data set of over nine million SNPs. Filtering based on mapping quality is a relatively simple and quick step in the bioinformatics process and removes reads aligning to multiple locations within the reference genome (Ribeiro et al., 2015). Even using mapping quality or a BLAST will not remove all false positive SNPs caused by misalignment, as Ribeiro et al. (2015) found the quality of the reference sequence to be the primary factor affecting false positive SNP generation and although the canola reference is largely complete, it is not perfect, with numerous ambiguous regions composed of N’s. As such, reads originating from one sub-genome may align to the other if only one of the homoeologous regions is present in the reference genome. Should this be the case, all reads arising from such a set of homoeologous regions would align to a single region in the reference and so pass mapping quality filtering and likely appear as heterozygous. Filtering for maximum heterozygosity of 10% removed 63% and 54% of SNPs from the GBS-t and WGR data, respectively (**Supplementary Methods S1**). Similarly, Cai et al. (2015) found 62% of SNPs to be heterozygous in a doubled haploid canola population, suggesting a large portion of false heterozygotes were removed in this study.

Completely filtering out false heterozygosity due to homoeology and other factors such as PCR errors while retaining highly heterozygous variants in a data set of this magnitude would be impractical, and in practice, the method used here is likely to remove the majority of false SNPs caused by homoeologous misalignment. Failing to adequately remove misalignments may affect downstream analyses, particularly LD estimates (**Supplementary Figure S2**), as SNPs arising from misalignments will be randomly spread throughout the genome and cause errors which will uniformly reduce LD. However, using stringent filtering parameters is likely to eliminate legitimately heterozygous SNPs, particularly newly arisen mutations which have not yet become fixed. As such, strict heterozygosity filtering will likely reveal more ancient and conserved patterns of LD.

Although the reference genome is the obvious target for improvement of canola genotyping, and ultimately a pan-genome should be considered, the development of a validated set of high-confidence SNPs and a curated list of SVs based on positions in the Darmor-*bzh* reference genome, would greatly ease the abovementioned issues and could be applied in data sets which would struggle to effectively apply the same measures. For example, SNPs identified in highly heterozygous crosses or hybrids could not be reliably filtered on heterozygosity, as a high degree of heterozygosity is expected. Using a set of previously validated SNPs, only loci which are known to be true SNPs could be analyzed. A variant database, such as has been established in Arabidopsis^3^, which includes not only position but also putative effects, subsequent amino acid changes and protein configuration would be of value to canola research communities.

The SNP list developed by Schmutzer et al. (2015) has made initial gains for *B. napus* in this area. Comparing the Schmutzer et al. (2015) 4.3-million SNP list and the c. 4 million high-confidence SNPs discovered in this study, 939,654 SNPs are present in both lists. As such, almost a quarter of the high-confidence SNPs identified in the present study have been independently verified. Perhaps a large proportion of SNPs are not in common due to the difference in the type of diversity represented in the two data sets. While the samples used in this study aim to represent diversity present in global oilseed breeding germplasm, the sample set used by Schmutzer et al. (2015) is representative of broader *B. napus* species diversity, including a large proportion of re-synthesized lines and vegetable types. As such, a significant proportion of loci from the Schmutzer et al. (2015) SNP list is expected to be of limited relevance to canola industry-based applications. The 149 varieties used in this study are expected to produce genomic resources of high relevance for industry-focused breeding and pre-breeding efforts. Furthermore, to ensure a high-confidence set of SNPs, extensive filtering was applied in the form of mapping quality, removing tri-allelic SNPs and low-confidence SNPs based on read depth, MAF, missing data, and heterozygosity.

As such, the SNP list developed in this study more fully represents genome-wide polymorphisms present in global breeding germplasm, and although this list needs to be expanded through the addition of validated SNPs from other types of canola, particularly additional Canadian and European spring types, this SNP list provides a common standard for application in other studies, and is a solid foundation for industry-oriented genomics within canola. Furthermore, the annotation of these high-confidence SNPs has allowed for broad characterization of SNP effects, and although these should be interpreted with caution, they provide a basis for further studies on gene effect and regulation, amino acid substitution, and effects on proteins and phenotypes.

The annotation of SNP effects broadly followed expectations with the majority of SNPs located in intergenic regions and a transition to transversion ratio of 1.3051, in line with previous findings in canola (Bus et al., 2012; Huang et al., 2013; Bayer et al., 2015). Somewhat unexpectedly, there were more non-synonymous than synonymous coding SNPs (57% versus 43%); however, other plant studies have found similar results, including 56% non-synonymous SNPs in oil palm (Pootakham et al., 2015), 57% in sorghum (Zheng et al., 2011), and 54–57% in rice (Subbaiyan et al., 2012; Jeong et al., 2013). Non-synonymous calls had a higher percentage of annotations with an error including multiple stop codons, transcript incomplete, and no-start codon (74% versus 71%), which could be due to incompleteness of the reference genome or the annotation of pseudogenes caused by polyploid functionalization of duplicated genes, in which case the presence of multiple stop codons is reasonable.

*Brassica napus* has undergone extensive duplication throughout its evolutionary history, which has resulted in a high degree of variability and adaptability. Allopolyploids are known to undergo diploidization after whole genome duplication, leading to gene loss and genomic SVs (reviewed by Fu et al., 2016). Whole genome sequences are a valuable resource for the evaluation of SVs such as the indels (<50 bp) identified by Mahmood et al. (2016) and Schmutzer et al. (2015), as well as the deletions and insertions (>50 bp) identified in the current study. The majority of SVs were found in non-coding regions although a significant number overlap genic regions, both coding and non-coding, confirming a functional role of SVs in gene effect and differentiation. Deletions have been previously associated with numerous agronomic traits such as zero erucic acid content (Wu et al., 2008), increased chlorophyll content (Qian et al., 2016) and seed glucosinolate content (Harper et al., 2012), and copy number variation in flowering genes has been linked to growth habit differentiation in canola (Schiessl et al., 2014, 2017).

Other chromosomal variants including inversions and duplications, which are difficult to identify using short-read sequencing, should also be investigated. In order to correctly identify repetitive regions including SVs, homoeologous regions, and repetitive elements, long-read sequencing and optical mapping will be required (reviewed by Fu et al., 2016). These approaches will also greatly assist with the improvement of the reference genome by preventing sequence collapse (Claros et al., 2012) and filling in missing gaps.

## Conclusion

The resources provided by sequencing the genomes of a large number of samples representative of global diversity is the foundation of a global, validated resource such as a variant database. While WGR is necessary to establish such resources, importantly, a low coverage reduced representation transcriptomics-based approach of GBS was shown to be sufficient to correctly identify clades in an NJ tree and was used for reliable side-by-side comparison in this study. As such, canola research should begin to focus on sequencing an increasing number of samples to truly take advantage of the diversity present in canola and increase the power of GS and GWAS.

## Author Contributions

MM prepared the plant materials and performed the sequencing library preparation. MM and FS performed the data analysis. MM, GS, HD, and NC all conceptualized the project and assisted in drafting the manuscript.

## Conflict of Interest Statement

The authors declare that the research was conducted in the absence of any commercial or financial relationships that could be construed as a potential conflict of interest.

## Footnotes

1 http://faostat3.fao.org/

2 https://www.ncbi.nlm.nih.gov/bioproject/

3 http://tools.1001genomes.org/polymorph/

## Supplementary Material

FIGURE S1 NJ tree showing distribution of varieties selected for WGR from GBS-t data.

FIGURE S2 LD plots based on WGR data with different maximum heterozygosity filtering applied.

FIGURE S3 STRUCTURE results for GBS-t data for K = 2.

FIGURE S4 Coverage of genome using covaris vs MspJI shearing methods.

TABLE S1 List of canola varieties used in the study.

TABLE S2 List of c. 4 million high-confidence SNPs provided in a VCF-style format.

TABLE S3 Average number of SVs identified by both programs and total number of unique SVs remaining after each filtering step.

TABLE S4 Size distribution of SVs.

METHODS S1 SNP filtering for excess heterozygosity and detection of ancestral populations using STRUCTURE.
