# Supplementary figures and images for: Diversity and Genome Analysis of Australian and Global Oilseed Brassica napus L. Germplasm Using Transcriptomics and Whole Genome Re-sequencing
Source: Front Plant Sci. 2018 Apr 19;9:508. doi: 10.3389/fpls.2018.00508 (PMC5917405; doi:10.3389/fpls.2018.00508)

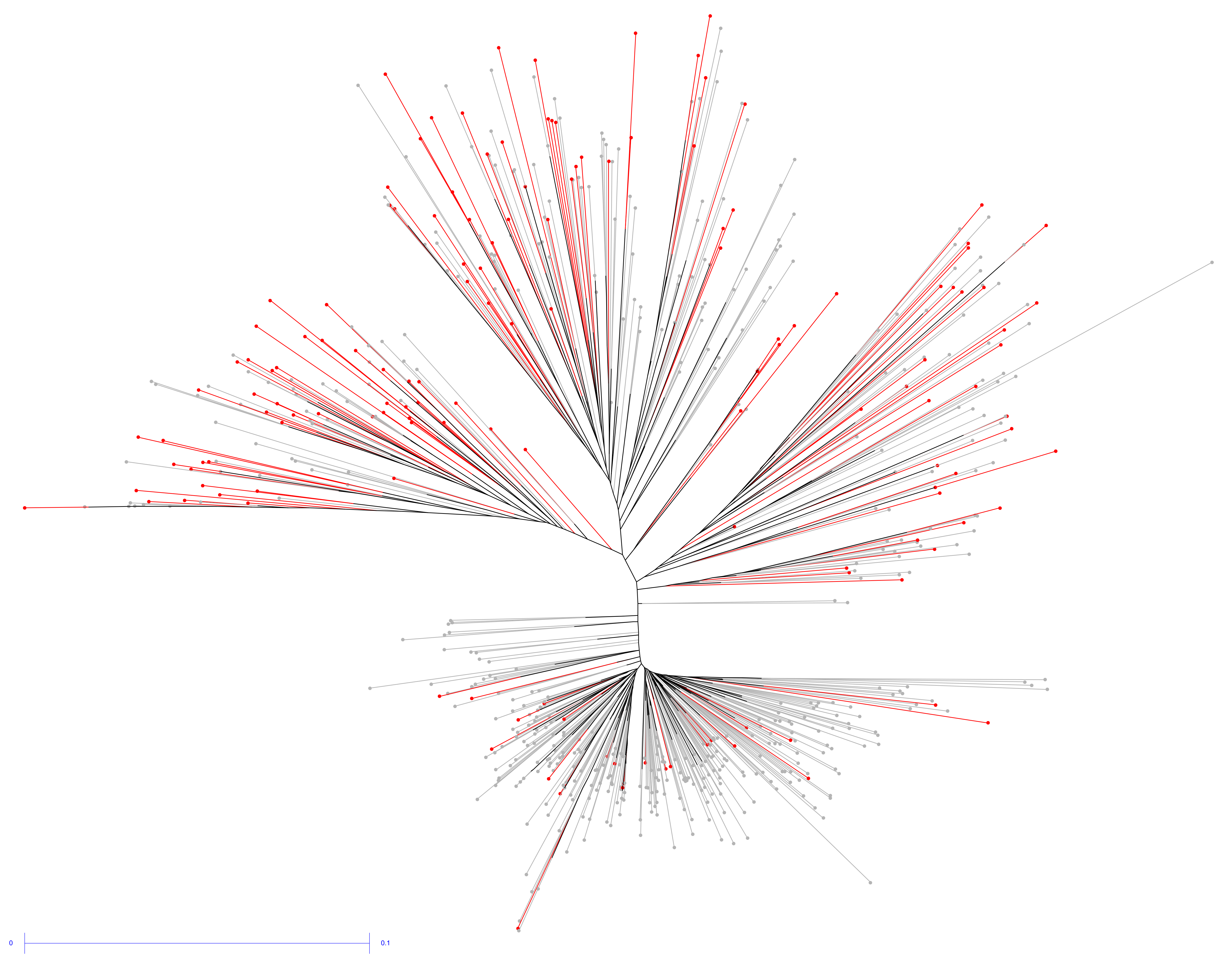

Supplement: FIGURE S1 — NJ tree showing distribution of varieties selected for WGR from GBS-t data. [file Image_1.TIF]

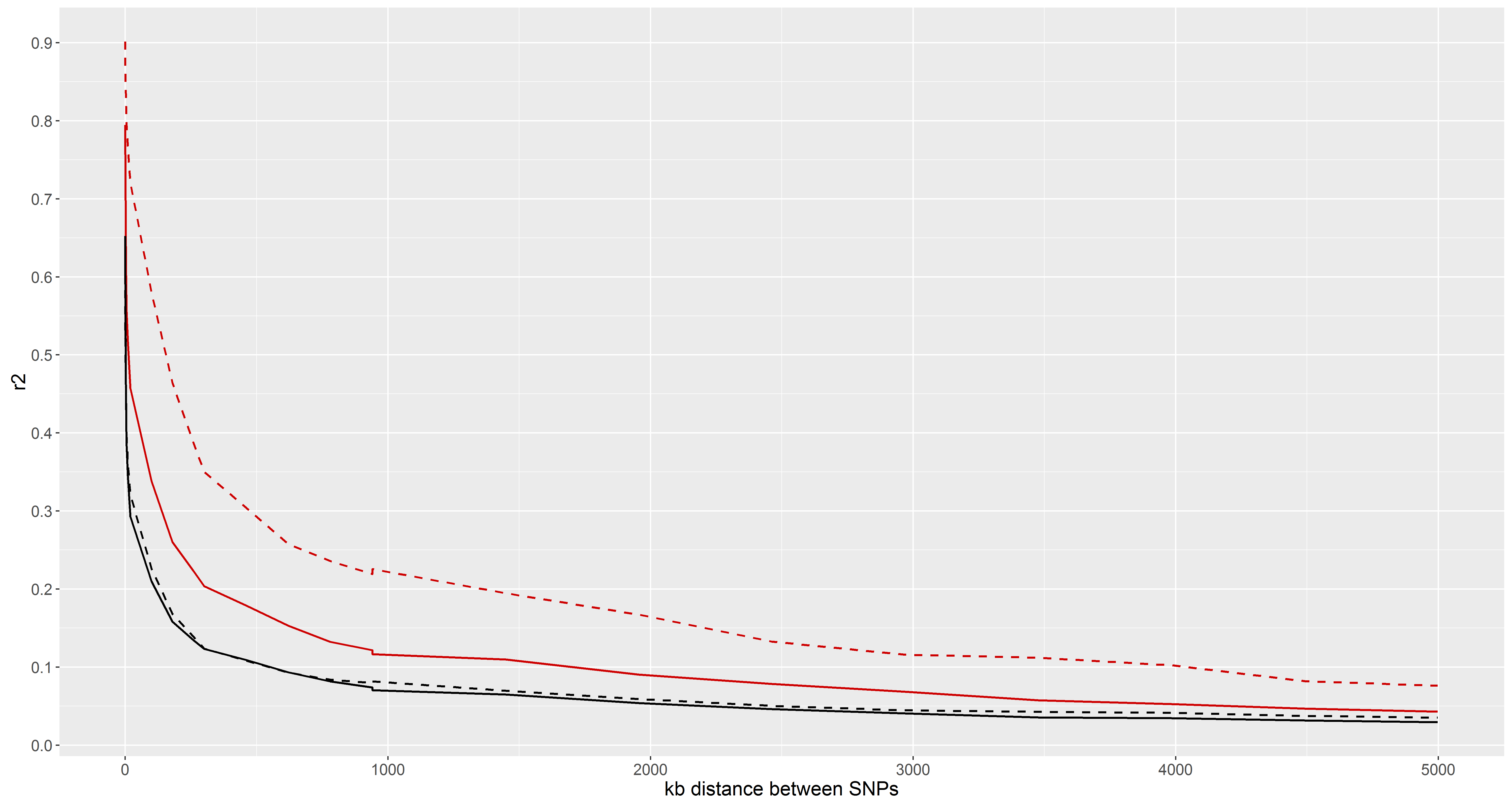

Supplement: FIGURE S2 — LD plots based on WGR data with different maximum heterozygosity filtering applied. [file Image_2.TIFF]

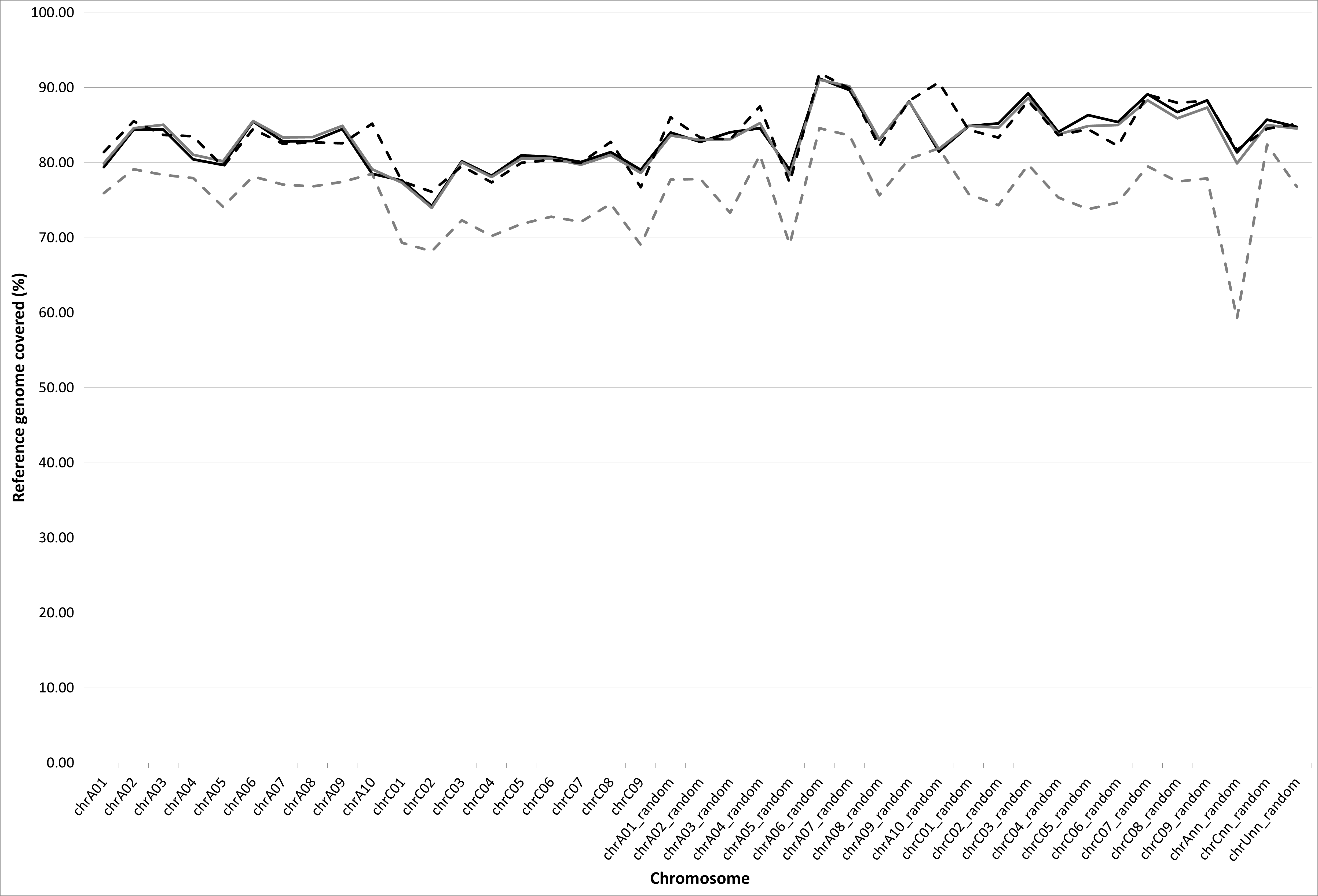

Supplement: FIGURE S4 — Coverage of genome using covaris vs MspJI shearing methods. [file Image_4.TIF]
