# Supplementary material for: Diversity and Genome Analysis of Australian and Global Oilseed Brassica napus L. Germplasm Using Transcriptomics and Whole Genome Re-sequencing
Source: Front Plant Sci. 2018 Apr 19;9:508. doi: 10.3389/fpls.2018.00508 (PMC5917405; doi:10.3389/fpls.2018.00508)
Supplement: METHODS S1 — SNP filtering for excess heterozygosity and detection of ancestral populations using STRUCTURE. [file Data_Sheet_1.DOCX]

***Supplementary Material***

**An assessment of global diversity and widespread whole genome sequencing for the establishment of genomic resources in oilseed *Brassica napus* L. germplasm**

**M.M. Malmberg, F. Shi, G.C. Spangenberg, H.D. Daetwyler, N.O.I. Cogan**

***Correspondence: noel.cogan@ecodev.vic.gov.au**

**1 Supplementary Methods**

***Heterozygosity filtering***

Due to the possibility of misalignment between homoeologous regions causing false positive SNPs, stringent filtering parameters were applied including minimum read depth, maximum missing data, minimum minor allele frequency as well as a minimum mapping quality of 30 and removing SNPs with excess heterozygosity. An initial examination of LD in the WGS data, applying minimum read depth of 5, maximum missing data of 40%, MAF of 0.1 across all samples and maximum heterozygosity filtering of either 40% or 10%, resulted in dissimilar LD profiles (Figure S2). There was little difference observed between the sub-genomes when filtering for maximum heterozygosity of 40%, while more stringent filtering of 10% resulted in an overall decrease in LD decay and greater differentiation between the sub-genomes, with LD decaying slower in the C genome.

As the effect of less stringent heterozygosity filtering on LD appears to be due to residual error, and the LD profile when applying stringent filtering is in line with expectations based on the results of previous studies, it was decided to use the more stringent heterozygosity filter of 10% across all data sets and for all analyses. This removed 63.4% and 54.4% of SNPs after other filters had already been applied.

***Identification of population structure using STRUCTURE***

To verify the population structure present in global canola germplasm as indicated by the neighbour-joining tree, the 633 GBS-t samples were processed with the software package STRUCTURE v2.3.4 (Pritchard et al., 2000). Additional SNP filtering was performed for this analysis. The c. 226K SNPs were filtered for minimum read depth of 5, maximum missing data of 40%, minimum MAF of 0.1 across all samples and maximum heterozygosity of 10%, resulting in 17,613 SNPs. The K number was determined by running five iterations for K=1 to K=6, with a burnin length of 10,000 and the same number of Monte Carlo Markov Chain replicates, under the admixture model. The most likely K number was determined with the method described by Evanno et al. (2005) using STRUCTURE HARVESTER (Earl, 2012). This indicated the most likely number of ancestral populations was K=2. The Q matrixes for the different runs were merged using CLUMPP (Jakobsson and Rosenberg, 2007) and plotted using *distruct* (Rosenberg, 2004).

The two ancestral populations likely represent spring and winter types as the majority of alleles in the Australian sub-population (0.87) are attributed to the first ancestral population (Q1), while the majority of alleles in the European sub-population (0.94) are attributed to the second ancestral population (Q2; Figure S3). Asian samples display almost equal contributions from both Q1 and Q2, which is consistent with a semi-winter growth habit. The samples of unknown origin are likely European winters based on a similar profile, with 0.89 of alleles derived from Q2, which is also consistent with their placement in the NJ tree (Figure 1).

**2 Supplementary Methods References**

Earl, D.A. (2012). STRUCTURE HARVESTER: a website and program for visualizing STRUCTURE output and implementing the Evanno method. *Conservation genetics resources* 4(2)**,** 359-361.

Evanno, G., Regnaut, S., and Goudet, J. (2005). Detecting the number of clusters of individuals using the software STRUCTURE: a simulation study. *Molecular ecology* 14(8)**,** 2611-2620.

Jakobsson, M., and Rosenberg, N.A. (2007). CLUMPP: a cluster matching and permutation program for dealing with label switching and multimodality in analysis of population structure. *Bioinformatics* 23(14)**,** 1801-1806.

Pritchard, J.K., Stephens, M., and Donnelly, P. (2000). Inference of population structure using multilocus genotype data. *Genetics* 155(2)**,** 945-959.

Rosenberg, N.A. (2004). DISTRUCT: a program for the graphical display of population structure. *Molecular Ecology Resources* 4(1)**,** 137-138.
